# Multiple extra-ischemic hemorrhages following intravenous thrombolysis in a patient with Trousseau syndrome: case study

**DOI:** 10.1186/s40064-015-0920-z

**Published:** 2015-03-25

**Authors:** Hiroyuki Ikeda, Rei Enatsu, Norikazu Yamana, Masaki Nishimura, Masaaki Saiki

**Affiliations:** Department of Neurosurgery, National Hospital Organization Himeji Medical Center, 68 Honmachi, Himeji City, Hyogo 670-8520 Japan

**Keywords:** Trousseau syndrome, Intravenous thrombolysis, Intracerebral hemorrhage, Cerebral infarction, Vascular disorders

## Abstract

**Background:**

Intracerebral hemorrhage is the most feared complication of intravenous thrombolysis for acute ischemic stroke. Such hemorrhage usually occurs within or at the margin of ischemic or manifestly infarcted brain tissue. A patient with Trousseau syndrome who developed multiple extra-ischemic hemorrhages following intravenous thrombolysis is described.

**Case description:**

An 80-year-old Japanese man with no other underlying disease was diagnosed with unresectable advanced lung cancer (stage IV) without brain metastasis and had not yet been treated. The patient suddenly presented with disturbance of consciousness, right hemiplegia, and total aphasia, and was admitted to our hospital. Magnetic resonance imaging revealed acute cerebral infarction extending from the basal ganglia to the corona radiata of the left cerebrum and multiple small areas of bilateral cerebral cortices. Cardiogenic cerebral embolism was considered among the differential diagnoses, but the brain natriuretic peptide level was within the normal range, and no arrhythmias such as atrial fibrillation were observed. With no other causes, the patient was diagnosed with Trousseau syndrome due to hypercoagulability associated with the advanced lung cancer. The patient received intravenous tissue plasminogen activator (t-PA) at 96 minutes after onset of symptoms. His symptoms partially improved, but they suddenly deteriorated at 84 minutes after the thrombolysis. A computed tomography (CT) scan immediately after the neurological deterioration revealed a subcortical hemorrhage in the left occipital lobe. A repeat CT scan the day after onset showed enlargement of the left occipital hemorrhage and two new subcortical hemorrhages in the right frontal and right temporal lobes. These hemorrhages were located in areas remote from the acute ischemic lesions.

**Conclusion:**

To the best of our knowledge, this is the first reported case of multiple extra-ischemic hemorrhages following intravenous thrombolysis in a patient with Trousseau syndrome. The course of this case suggests that intravenous t-PA administration for acute ischemic stroke with Trousseau syndrome may be associated with a higher risk of intracranial hemorrhage.

## Background

Although treatment with intravenous tissue plasminogen activator (t-PA) improves clinical outcomes in carefully selected patients with acute ischemic stroke, intracerebral hemorrhage is the most feared complication of this treatment (No authors 1995). Incidence rates of symptomatic intracranial hemorrhage have been reported to account for 5-20% of acute ischemic strokes following intravenous thrombolysis in some clinical studies (Albers et al. 2000; Hill and Buchan 2005; Wahlgren et al. 2007). The majority of such hemorrhages occur within or at the margin of ischemic or manifestly infarcted brain tissue (Wahlgren et al. 2007). Meanwhile, intracerebral hemorrhage can also appear in brain tissue without apparent acute ischemia, and the incidence of extra-ischemic hemorrhages has been reported at 1.3-3.7% of acute ischemic stroke following intravenous thrombolysis (No authors 1997; Trouillas and von Kummer 2006; Wahlgren et al. 2007). Intra- and extra-ischemic hemorrhages are assumed to have at least partly different underlying pathophysiological mechanisms (Mazya et al. 2014).

In 1865, Trousseau described migratory thrombosis as the first manifestation of occult gastric cancer (Rickles and Edwards 1983). Since then, the association between cancer and excessive blood coagulation has remained well-recognized. The description of Trousseau syndrome was refined and reported as being frequently associated with chronic disseminated intravascular coagulation, plate-rich microthrombi, macroangiopathic hemolytic anemia, verrucous endocarditis, and thromboembolic problems related to these processes (Sack et al. 1977). Uchiyama et al. described that Trousseau syndrome is a paraneoplastic neurologic syndrome caused by remote effects of occult cancers, which causes neurological symptoms due to hypercoagulability associated with cancers (Uchiyama and Shimizu 2003). Today, the concept of Trousseau syndrome is commonly used not only to describe migratory thrombosis that precedes the diagnosis of occult cancer, but also any hypercoagulability associated with malignant cancer (Varki 2007). Thus, when acute ischemic strokes occur in patients with cancer, Trousseau syndrome should be considered in the differential diagnosis. In the current American Heart Association/American Stroke Association guidelines, intravenous t-PA administration in acute ischemic stroke associated with Trousseau syndrome is not mentioned (Jauch et al. 2013). To the best of our knowledge, this is the first reported case of multiple extra-ischemic hemorrhages following intravenous t-PA in a patient with Trousseau syndrome.

## Case description

**Figure 1:**
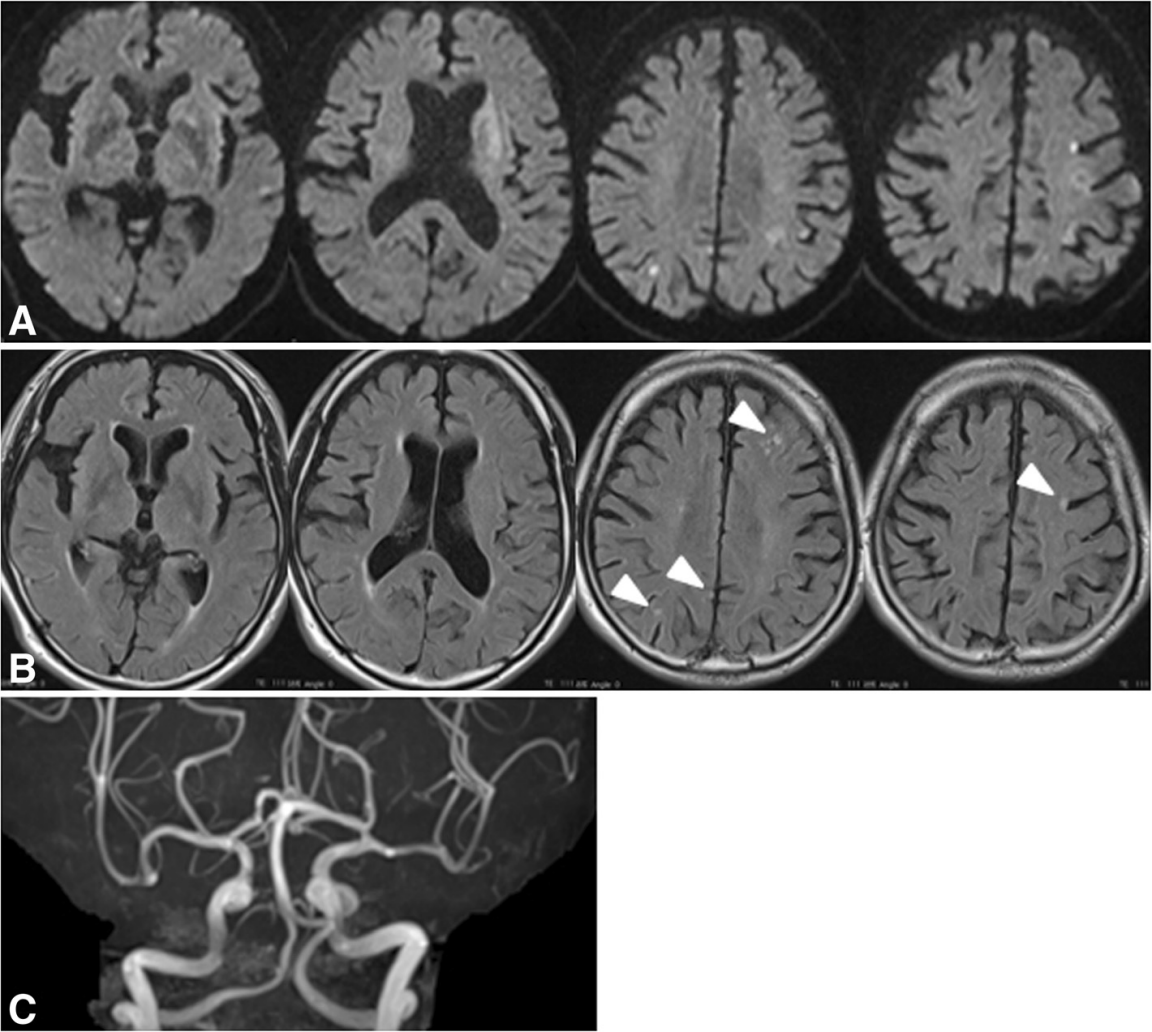
**Head magnetic resonance imaging after onset. A:** Diffusion-weighted image (DWI) showing a high-intensity area extending from the basal ganglia to the corona radiata of the left cerebrum and multiple small high-intensity areas at bilateral cerebral cortices. **B:** Fluid-attenuated inversion recovery showing several small high-intensity areas in the same locations detected on DWI (arrowheads). **C:** Magnetic resonance angiography demonstrating no steno-occlusive changes.

An 80-year-old Japanese man with no other underlying disease had a chief complaint of cough, and chest radiography showed an abnormal shadow in the right lung. He was referred to our hospital for diagnosis and treatment. A 9-cm lesion was observed in the right lower lobe on contrast-enhanced computed tomography (CT) of the chest. Transbronchial lung biopsy showed this to be adenocarcinoma 24 days before the onset of cerebral infarction. Metastases to ipsilateral hilar lymph nodes, liver, and the left kidney were suggested by contrast-enhanced CT of the trunk, and he was determined to have stage IV advanced lung cancer. Brain metastasis was not detected on head gadolinium-enhanced magnetic resonance imaging (MRI) 15 days before the onset of cerebral infarction. Since a curative operation was impossible, he was scheduled to be treated with systemic chemotherapy. However, he developed a sudden disturbance of consciousness, right hemiplegia, and total aphasia while walking outdoors, and he was admitted to our hospital 32 minutes after the onset of symptoms. His temperature was 36.8°C, blood pressure was 125/68 mmHg, and heart rate was 82 beats/min with no atrial fibrillation. His initial National Institutes of Health Stroke Scale (NIHSS) score was 26. His complete blood counts were as follows: white blood cell count 5400 /μL, red blood cell count 3,520,000 /μL, hemoglobin 10.8 g/dL, and platelets 196,000 /μL. The coagulation system showed activation, with a fibrinogen level of 629 mg/dL (normal 200–400 mg/dL), a D-dimer level of 6.02 μg/mL (normal: 0–0.99 μg/mL), and a fibrin degradation product level of 15 μg/mL (normal: 0–5 μg/mL), with a prothrombin time of 13.6 s (normal: 10–12 s; prothrombin time international ratio of 1.15) and an activated partial thromboplastin time of 32.5 s (normal: 24–39 s). Cardiogenic cerebral embolism was considered in the differential diagnosis, but the brain natriuretic peptide level was 17.2 pg/mL (normal: 0–18.4 pg/mL), which was within the normal range. The other blood biochemical test results were not abnormal except for elevated C-reactive protein (8.73 mg/dL). He was not diagnosed with disseminated intravascular coagulation based on the clinical and laboratory information. Chest radiography did not show any enlargement of the superior mediastinal shadow or cardiac shadow. Diffusion-weighted imaging (DWI) showed a high-intensity area extending from the basal ganglia to the corona radiata of the left cerebrum and multiple small high-intensity areas at bilateral cerebral cortices (Figure 1A). Among these acute ischemic lesions, some showed hyper-intensity signals on fluid-attenuated inversion recovery (FLAIR) imaging (Figure 1B). T2*-weighted imaging did not demonstrate hypointense signal areas or spots that suggested cerebral microbleeds. Magnetic resonance angiography (MRA) demonstrated no steno-occlusive changes in cerebral arteries (Figure 1C).

**Figure 2:**
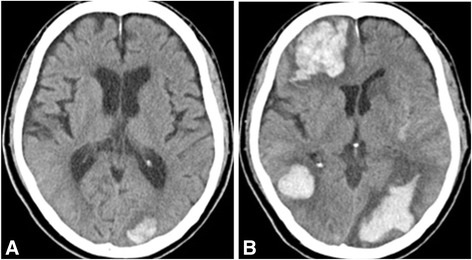
**Head computed tomography (CT) scans after thrombolysis. A:** CT scan right after the neurological deterioration showing a subcortical hemorrhage in the left occipital lobe. **B:** Repeat CT scan the day after onset showing enlargement of the left occipital hemorrhage and two subcortical hemorrhages in the right frontal and right temporal lobes.

Although the long-term prognosis of the lung cancer was considered poor, the patient received 0.6 mg/kg of intravenous t-PA starting 96 minutes after onset of symptoms because of no identifiable contraindications. His symptoms improved partially (NIHSS score: 18), but he suddenly became stuporous at 84 minutes after the intravenous t-PA (NIHSS score: 25). Blood pressure was 134/72 mmHg with a heart rate of 76 beats/min at the time. A CT scan immediately after the neurological deterioration showed a subcortical hemorrhage in the left occipital lobe (Figure 2A). A repeat CT scan the day after onset showed enlargement of the left occipital hemorrhage and two new subcortical hemorrhages in the right frontal and right temporal lobes (Figure 2B). These three intracerebral hemorrhages were located in extra-ischemic areas that were remote from the acute ischemic lesions detected on DWI. The patient was given conservative treatment for the intracranial hemorrhages due to the poor prognosis for the primary disease. Fourteen days of electrocardiographic monitoring conducted after the ischemic stroke did not show arrhythmias such as atrial fibrillation. Transthoracic echocardiography and venous ultrasonography of the lower limbs were conducted to search for the source of embolism, but did not show any abnormalities. Despite the presence of hypercoagulability and suspected cerebral embolism, neither source of embolism nor obvious factor was found. Therefore, he was diagnosed as having Trousseau syndrome associated with advanced lung cancer. Systemic chemotherapy and anticoagulant therapy were not performed. He was discharged home 66 days after admission with a modified Rankin Scale score of 5, and he died due to the primary disease 136 days after the ischemic stroke. The autopsy was not performed.

## Discussion and evaluation

There are many causes of thrombosis in Trousseau syndrome associated with cancer because the pathogenesis of Trousseau syndrome is complex; cancer itself often causes the underlying mechanism. When cells of the monocyte or macrophage lineage interact with malignant cells, they release tumor necrosis factor, interleukin-1, and interleukin-6. These cytokines cause endothelial damage and sloughing of endothelial cells and thereby promote thrombosis (Bick 2003). The interaction between tumor cells and macrophages also activates platelets, factor VII, and factor X, which leads to the generation of thrombin and thrombosis (Bick 2003). Trousseau syndrome often occurs with mucinous adenocarcinomas such as lung, pancreatic, and ovarian cancers (Sutherland et al. 2003). Circulating carcinoma mucins interacting with leukocyte L-selectin and platelet P-selectin generate platelet aggregation without requiring accompanying thrombin generation (Wahrenbrock et al. 2003). Mucins from adenocarcinomas also cause nonenzymatic activation of factor X (Bick 2003).

Treatment with intravenous t-PA for acute ischemic stroke is associated with an increased risk of intracerebral hemorrhage. Symptomatic intracerebral hemorrhages following intravenous thrombolysis are associated with several factors, including older age, increased stroke severity, higher glucose level, prior antiplatelet use, presence of atrial fibrillation, congestive heart failure, renal impairment and extent of early ischemic changes (Dzialowski et al. 2006; Whiteley et al. 2012). In particular, delayed intravenous t-PA is associated with a higher incidence of intracranial hemorrhage, so earlier treatment is recommended (Lees et al. 2010). In the present case, the patient developed intracerebral hemorrhages 84 minutes after the intravenous t-PA, strongly suggesting an association between the hemorrhages and the thrombolytic therapy. Although the exact pathogenesis of intracerebral hemorrhages following intravenous t-PA in a patient with Trousseau syndrome remains unknown, there are at least two possible mechanisms from the findings in the present patient. First, initial MRI showed several FLAIR hyperintensities among the acute ischemic lesions on DWI, suggesting mixed ischemic lesions at different developmental times. Development of FLAIR hyperintensity within an acute ischemic lesion on DWI is associated with an increased risk of hemorrhagic transformation after thrombolysis (Kufner et al. 2013). Therefore, the present patient may have had a potential risk of hemorrhagic transformation at the FLAIR hyperintensities. Second, the present patient developed cerebral hemorrhages in regions without visible ischemic tissue changes. These extra-ischemic hemorrhages suggest pre-existing brain pathology, especially cerebral amyloid angiopathy (Trouillas and von Kummer 2006), and cerebral microbleeds are related to cerebral amyloid angiopathy and hypertensive microangiopathy (Vernooij et al. 2008). Therefore, the present patient without microbleeds on MRI had a lower risk of amyloid angiopathy-associated hemorrhages. So why did the present patient with Trousseau syndrome develop extra-ischemic hemorrhages? Although the old age and great stroke severity may be associated with intracranial hemorrhages following thrombolysis, we think the other factors are also associated with intracranial hemorrhages due to mulitiple and extra-ischemic hemorrhages immediately after thrombolysis. We hypothesize that micro-thromboembolism, micro-metastasis, or micro-tumor embolism, which cannot be detected on MRI, at areas remote from the acute ischemic lesions may have caused extra-ischemic hemorrhages (Bokuda and Nozaki 2012). This suggests that intravenous t-PA administration for acute ischemic stroke with Trousseau syndrome may be associated with potential risks of intracranial hemorrhage both in the ischemic lesions and in brain areas remote from the infarcted tissue.

Some studies have compared intracerebral hemorrhage rates following thrombolytic therapy for acute ischemic stroke between patients with and without cancer, and patients with cancer do not appear to have an increased risk of intracranial hemorrhage (Masrur et al. 2011; Murthy et al. 2013). However, there was no information on coagulopathy in the study by Masrur et al., and only 40 (5.0%) of the 807 patients with cancer had coagulopathy in the study by Murthy et al. Therefore, these studies are not relevant to patients with a cancer-associated hypercoagulable state (Trousseau syndrome). Little is known about the risk of intracerebral hemorrhage following thrombolysis in patients with Trousseau syndrome because these patients generally have been excluded from most clinical trials.

D-dimer levels are used in many studies as a direct indicator of hypercoagulability (Grisold et al. 2009). A cancer-associated hypercoagulable state represents a risk factor for cerebral infarction, and D-dimer levels in cancer patients have been reported as significantly higher in patients with cerebral infarction due to cryptogenic stroke mechanisms compared to conventional stroke mechanisms (Kim et al. 2010; Schwarzbach et al. 2012). In particular, cancer patients with metastatic lesions are known to show significantly increased D-dimer levels, and are significantly more likely to develop cerebral infarction due to cryptogenic stroke mechanisms (Kim et al. 2010; Hejna et al. 1999). Thus, advanced cancer patients with cryptogenic stroke with activation of the D-dimer level may be a predictor of the diagnosis of Trousseau syndrome. Therefore, careful confirmation with or without advanced cancer, cryptogenic stroke, and activation of the D-dimer level may be needed before the decision to administer intravenous thrombolysis.

Although the present patient had no identifiable contraindications to intravenous thrombolysis, perhaps thrombolytic therapy should not have been used. We publish this case report, due to the showing the attention of unexpected extra-ischemic hemorrhages associated with Trousseau syndrome despite the wrong decision on using thrombolytic therapy in this patient. Clear guidelines are lacking on intravenous t-PA administration for acute ischemic stroke with Trousseau syndrome, and further investigations and analyses of more case reports are needed.

## Conclusion

This is the first known case report of multiple extra-ischemic hemorrhages following intravenous t-PA in a patient with Trousseau syndrome. The course of the present case suggests that intravenous t-PA administration for acute ischemic stroke with Trousseau syndrome may be associated with a higher risk of intracerebral hemorrhage.

## Consent

Written informed consent was obtained from the wife of the patient for publication of this case report and any accompanying images. A copy of the written consent is available for review by the Editor of this journal.

## Abbreviations

t-PA: Tissue plasminogen activator
CT: Computed tomography
MRI: Magnetic resonance imaging
NIHSS: National Institutes of Health Stroke Scale
DWI: Diffusion-weighted imaging
FLAIR: Fluid-attenuated inversion recovery
